# Telomere and Telomerase Therapeutics in Cancer

**DOI:** 10.3390/genes7060022

**Published:** 2016-05-26

**Authors:** Yucheng Xu, Amir Goldkorn

**Affiliations:** Division of Medical Oncology, Department of Medicine, Keck School of Medicine and Norris Comprehensive Cancer Center, University of Southern California, Los Angeles, CA 90033, USA; yuchengx@usc.edu

**Keywords:** telomeres, telomerase, targeted therapy

## Abstract

Telomerase is a reverse transcriptase capable of utilizing an integrated RNA component as a template to add protective tandem telomeric single strand DNA repeats, TTAGGG, to the ends of chromosomes. Telomere dysfunction and telomerase reactivation are observed in approximately 90% of human cancers; hence, telomerase activation plays a unique role as a nearly universal step on the path to malignancy. In the past two decades, multiple telomerase targeting therapeutic strategies have been pursued, including direct telomerase inhibition, telomerase interference, hTERT or hTERC promoter driven therapy, telomere-based approaches, and telomerase vaccines. Many of these strategies have entered clinical development, and some have now advanced to phase III clinical trials. In the coming years, one or more of these new telomerase-targeting drugs may be expected to enter the pharmacopeia of standard care. Here, we briefly review the molecular functions of telomerase in cancer and provide an update about the preclinical and clinical development of telomerase targeting therapeutics.

## 1. Introduction

In January 2016, President Barack Obama issued a “Moonshot” memorandum deploying a task force led by Vice President Joseph Biden with the goal of accelerating cancer prevention and treatment. In this renewed search for novel, high impact strategies, telomerase continues be pursued as a promising therapeutic target and biomarker. The Nobel Prize in physiology or medicine of 2009 was awarded to Elizabeth H. Blackburn, Carol W. Greider, and Jack W. Szostak for their discovery of telomerase, which plays a key role in the formation and progression of up to 90% of malignancies. In this review, we describe the biology of telomeres and telomerase in cancer, as well as the current telomerase based therapeutics, some of which may enter clinical use in the coming years.

## 2. Telomere Biology

Telomeres are short tandem repetitive DNA sequences that are located at the ends of chromosomes. Telomeres contain double-stranded TTAGGG DNA with a single stranded 3’ telomeric overhang that loops back and invades the duplex telomeric region, disrupting the double helix and base-pairing to one of the two strands [1]. Telomeres play a “capping” function by protecting the ends of chromosomes from degradation and fusion. A group of telomere associated proteins contribute to this protective function; collectively dubbed shelterin proteins, they include telomere repeat factor 1 (TRF1), telomere repeat factor 2 (TRF2), protection of telomere 1 (POT1), repressor/activator protein 1 (RAP1), TRF1-and TRF2-interacting nuclear protein 2 (TIN2), and TINT1/PTOP/PIP1 protein (TPP1) [2]. TRF1, TRF2, and POT1 directly bind to telomeric repeats, with TRF1 and TRF2 binding to double-stranded DNA [3,4], and POT1 binding to single-stranded overhangs [5]. It was originally observed that hRap1 was recruited to telomeres by hTRF2 [6], but more recently hRap1 was also found to be capable of directly binding telomeric DNA (albeit at lower affinity) and regulating hTRF2 recruitment [7,8]. TIN2 binds TRF1 and TRF2 through protein-protein interactions with distinct domains [9], but TPP1 does not bind directly to telomeric DNA, as it interacts and forms a complex with TIN2 and POT1 [10,11]. 

In normal somatic cells, telomere shortening occurs at each chromosome replication step due to the well-recognized “End Replication Problem”, wherein spaces occupied by the RNA primer at the end of the lagging strand during *S* phase are not replenished, losing 50–200 nucleotides at each cycle [12,13]. Telomere shortening continues until replicative senescence is triggered when the length of telomeres is about 4–6 kb, also known as mortality stage 1 (M1). However, some cells manage to bypass M1 by inactivating cell-cycle checkpoint pathways (e.g., p53 and or p16/RB) and continue to shorten, eventually entering mortality stage 2 (M2 or crisis), characterized by genomic instability, fusion/breakage mutagenic events, and massive cell death. Very rarely, some cells can reactivate/upregulate telomerase that is absent in most normal somatic cells at M1 or M2 to stabilize telomere length, leading to immortalization. Although immortalization is not sufficient to induce malignant transformation, immortalization acquired from activated telomerase in combination with genome instability and mutation from telomere shortening potentiates cancer formation [14].

It was originally thought that telomeres are transcriptionally silent until the discovery of telomeric repeat-containing RNA (TERRA) [15]. TERRA is transcribed by DNA-dependent RNA polymerase II from the subtelomeric regions toward the chromosome ends and composed of subtelomeric and telomeric repeats [16]. Once transcribed, TERRA localizes to chromosome ends where it either interacts with shelterin components like TRF1 and TRF2 or base-pairs with its template DNA strand to form RNA:DNA hybrid structures known as R-loops [16,17]. TERRA has been reported to participate in the DNA damage response (DDR) triggered by deprotected telomeres by interacting with the histone methyltransferase SUV39H1 and promoting methylation of histone H3K9 at damaged telomeres upon TRF2 depletion [18]. The 3’ end of TERRA is complementary to the template region of telomerase RNA TERC, and *in vitro* assays demonstrated that TERRA is a natural ligand and direct inhibitor of telomerase [19]. However, the role of TERRA as a negative regulator of telomerase is still being confirmed by further *in vivo* studies.

## 3. Telomerase Biology

Telomerase is a ribonucleoprotein responsible for maintaining telomere length. The core of telomerase has two components: Catalytic telomerase reverse transcriptase (TERT) and telomerase RNA (TERC). TERT utilizes the template region (3’-CAAUCCCAAUC-5’) of TERC to add TTAGGG DNA repeats and thereby extend single stranded 3’ telomeric strands [20]. In addition to these two core components, several accessory proteins associate with the telomerase holoenzyme, including telomerase cajal body protein 1 (TCAB1) [21], the four H/ACA-motif RNA binding proteins dyskerin [22], NHP2, NOP10, Gar1 [23], and the two ATPase proteins pontin and reptin [24].

Aberrations in telomerase and its associated proteins have been linked with disease. For instance, dyskeratosis congenita (DKC), a syndrome characterized by a classic triad of nail dysplasia, skin pigmentary changes, oral leukoplakia, and bone marrow failure, is associated with short telomeres and mutations in TCAB1 [25], dyskerin [26], NHP2 [26], and NOP10 [27]. Telomerase reactivation by recurrent somatic mutations in the TERT promoter has been identified in cancers of the central nervous system (43%), bladder (59%), thyroid (10%), and melanoma (29%) [28].

In clinical studies, aggressive metastatic disease and poor prognosis have been correlated with high telomerase expression and activity in ovarian, breast, and colorectal cancers and melanoma [29,30,31,32]. Our group has detected high telomerase activity in live captured circulating tumor cells (CTCs), and we found that higher activity was predictive of shorter survival in prostate cancer patients [33,34]. Others have described a direct association between therapy resistance and telomerase expression and activity in breast and gastric cancer patients [35,36]. Parallel *in vitro* studies have provided preliminary evidence supporting a direct role for telomerase in drug resistance: Ectopic overexpression of TERT in cancer cell lines rendered them less sensitive to radiation and imatinib [37,38]. Similarly, acquired chemotherapy resistance in various cell lines was associated with TERT upregulation, whereas attenuation of telomerase activity resensitized cell lines to various chemotherapeutics [39,40,41]. Drug resistance and tumor progression have been attributed to subpopulation of cancer cells, termed cancer stem cells (CSC), and telomerase has been implicated in the potentiation of these cells. For example, ectopic expression of TERT has been shown to potentiate CSC properties in breast cancer cells *in vitro* [42], whereas telomerase inhibition was shown to deplete CSC in pancreatic, prostate, lung, breast, and glioma cell lines [43,44,45,46]. In our own work, we showed that subpopulations of cells with CSC properties isolated from freshly resected prostatectomies had extremely high telomerase expression and activity levels relative to the rest of the tumor cells, and that targeting telomerase could effectively neutralize these subpopulations [47].

Recently, several non-canonical oncogenic functions of telomerase, independent of its role in direct telomere maintenance, have been described [48]. Ectopic expression of telomerase activated NF-κB dependent genes such as IL-6, TNF-α, MCP1 and IκBα, while telomerase-null mice were defective in response to NF-κB signaling. TERT was found to bind to a NF-κB p65 subunit through protein-protein interaction to form a protein complex, which was subsequently recruited to a set of NF-κB target gene promoters. Interestingly, telomerase as a core holoenzyme had much better capacity to activate NF-κB pathway than TERT or TERC alone [49], suggesting that both contributed to these non-canonical roles. In another study, catalytically dead TERT physically interacted with the NF-κB p65 subunit, promoting p65 nuclear localization and DNA binding, and enhancing NF-κB mediated transcription of several matrix metalloproteinase, e.g., MMP-1, -3, -9,-10 [50]. Expression of telomerase protein (TERT) in fibroblasts which had been immortalized with SV40 and H-Ras rendered the cells highly tumorigenic, an effect equally conferred by TERT-HA, a variant that was incapable of elongating telomeres *in vivo* [51]. Additional work in normal tissue stem cells and in cancer cells suggested that telomerase may exert a non-canonical role that also involves signaling through direct interaction with β-catenin and activation of transcriptional target genes [52,53,54,55,56,57]. Most recently, it has also been demonstrated that TERT, but not TERC, regulates MYC dependent oncogenesis, suggesting that TERT functions as a regulator of MYC without TERC and hence, independent from its reverse transcriptase activity [58]. This study also was the first to genetically dissect the role of TERT versus TERC in regulating MYC-driven oncogenesis *in vivo* because TERT knockout mice, but not TERC knockout mice, had delayed onset of MYC-driven lymphomagenesis [58].

Recent findings have also assigned a non-canonical function to telomerase RNA (TERC), albeit not as yet in human models. Genetic deletion of TERC in zebrafish caused impaired myelopoiesis including neutropenia and monocytopenia, despite the fact that the telomere length was unchanged [59]. TERC regulates myelopoiesis, and controls the fate of myeloid vs erythroid of haematopoietic stem cells (HSCs) by differentially regulating the levels of the master myeloid and erythroid transcription factors spi1 and gata1a, respectively [60]. Another study showed that a small transposable element in *Arabidopsis thaliana*, in response to DNA damage, can translocate into a TERC isoform TER2, resulting in modulation of telomerase activity [59].

## 4. Telomerase as a Cancer Biomarker

Telomerase activity is temporally regulated in accordance with developmental requirements. This activity is detected at fetal and newborn stages, but gradually declines to extremely low or no activity from the neonatal period onward, with the exception of highly regenerative cells including hematopoietic, epidermal, and gastrointestinal cells [61,62]. In contrast, telomerase activity is highly upregulated in most of cancers that have been surveyed across in the vast majority of malignancies [63], including breast [30,64], colon [31,65], melanoma [32], oral [66,67], ovarian [29], pancreas [68], prostate [69], and soft tissue cancer [70]. This distinct phenotypical disparity between normal/benign and malignant tissues creates the potential for telomerase to serve as a universal cancer biomarker for disease diagnosis and prognosis.

In 1994, a method dubbed TRAP (telomeric repeat amplification protocol) was developed to measure telomerase activity. This assay consists of two steps, an extension reaction in which a “telomerase substrate” (TS) oligonucleotide mimicking a 3’ telomere end is combined with cell lysate containing telomerase, and an amplification reaction in which any TS oligo extended by telomerase in the lysate is PCR amplified for detection [71]. This highly sensitive assay can detect as few as 1–10 telomerase positive cells or 0.01% positive cells in a mixed population [63]. Using TRAP, telomerase activity has been identified in the vast majority of cancer types but not in selected normal somatic tissues although TRAP activity was detected in germ-line tissues and weak activity was found in peripheral blood leukocytes and in certain stem cell populations [72].

In addition to being found in primary tumor, telomerase activity has been identified in circulating tumor cells (CTCs). In breast cancer, CTC telomerase activity was detected in 21 of 25 (84%) patients but was undetectable from nine healthy donors [73]. In ovarian cancer, telomerase activity was identified in CTCs of all stage IV patients (100%), but none of the healthy volunteers [74]. In a phase III trial for men with metastatic hormone sensitive prostate cancer, we showed that in patients with five or more CTCs per 7.5 ml of blood, CTC telomerase activity was prognostic of overall survival (hazard ratio 1.14 with *P* = 0.001 [75].

Beyond telomerase enzymatic activity, TERT mRNA expression level has been studied as biomarker, because it has been demonstrated to be the rate-limiting determinant of telomerase activity in various malignancies, such as urothelial cancer [76], non-small cell lung cancer [77], and breast cancer [78]. Plasma TERT mRNA from prostate cancer patients was demonstrated as a valid diagnostic and prognostic tool with 85% sensitivity, 90% specificity, 83% positive predictive value, and 92% negative predictive value [79]. Another study in 234 cancer patients with 28 different types of cancer showed the patients’ serum TERT mRNA levels correlated with the clinical parameters of metastasis and recurrence (*P* < 0.001) [80]. In contrast to TERT mRNA, telomerase RNA, TERC, is generally expressed ubiquitously, at times in the absence of telomerase activity [81], and thus it has been assumed that TERC would not be a suitable cancer marker. However, significantly increased TERC RNA levels were found in astrocytomas [82], gastric carcinomas [83], and oesophageal cancer [84]. In breast cancer, TERC was detected by real-time PCR (qPCR) in 17 of 18 tumors (94%), but was undetectable in benign tissues [85]. Recently, telomerase activity, TERT mRNA, and TERC telomerase RNA were measured in urine of bladder cancer patients with various sensitivities and specificity rates [76,86,87]. A study from 200 bladder patients comparing these three methods in urine revealed that sensitivity f of TERT mRNA, TERC, and telomerase activity for detecting bladder cancer was 96%, 92%, and 75%, respectively [88].

## 5. Telomere and Telomerase Therapeutics

**Figure 1:**
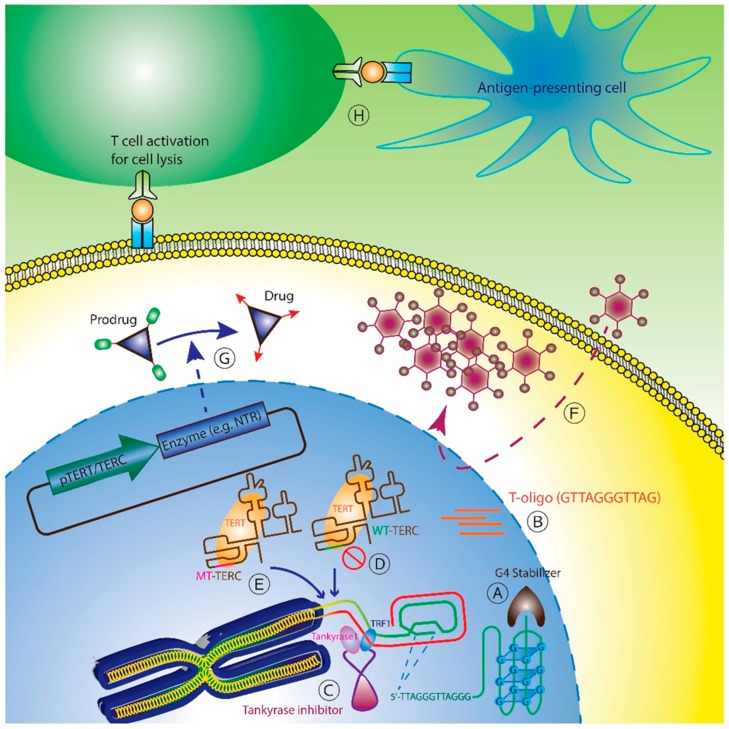
Overview of telomerase therapeutic strategies. Several telomerase-based therapeutic approaches have been developed: (**A**) G-quadruplex stabilizers prevent unwinding of the quadruplex, making the 3’ overhang inaccessible to telomerase; (**B**) Telomeric oligos (T-oligo) mimic uncapped telomeres and elicit a DNA damage response; (**C**) Tankyrase inhibitors inhibit dissociation of TRF1 from telomeres, rendering them less accessible to telomerase; (**D**) Direct inhibition of telomerase enzymatic activity via an oligonucleotide (Imetelstat) that binds TERC template; (**E**) Telomerase interference (MT-TERC) that reprograms telomerase to add mutant telomeric repeats that elicit a DNA-damage response; (**F**) TERT promoter driven oncolytic viruses that cause tumor-specific cell lysis; (**G**) Suicide gene therapy that employs TERT or TERC promoter-driven expression of enzymes that convert pro-drugs to active cytotoxic molecules; (**H**) Telomerase vaccines that induce cytotoxic T lymphocyte by either direct inoculation or *ex vivo* activation.

High telomerase expression and activity in cancer cells, compared to low levels of telomerase observed in very few adult stem cell compartments, such as hematopoietic, epidermal, and gastrointestinal cells, have fueled significant efforts to target telomerase therapeutically. Several strategies are currently in various stages of development and are discussed based on mechanism of action: (1) Telomere-based strategies; (2) Telomerase interference; (3) Direct telomerase inhibition; (4) TERT or TERC promoter driven therapy; and (5) Telomerase vaccines (Figure 1).

### 5.1. Telomere-Based Strategies

Telomere-based approaches offer a potential advantage in that they directly mimic or interfere with telomere structures rather than disrupting telomerase. Hence, these strategies may be effective even for telomerase-negative malignancies (e.g., sarcomas, some lymphomas) that maintain their telomeres using alternative lengthening of telomeres (ALT, a homologous recombination-based telomere-elongation mechanism [89].

G-quadruplex stabilizers: G-quadruplex is a tetrad planar structure formed by four guanine bases stabilized by Hoogsteen hydrogen bonds, and this structure can be formed in guanine rich nucleic acids including telomeres [90,91]. The putative function of G-quadruplex structures is to protect telomere ends from nuclease attack [92]. These protective quadruplex configurations are recognized and partially unwound by telomerase for 3’ end extension [93]. Conversely, binding of G-quadruplex structures by so-called “stabilizers” effectively “locks” the telomeres in the quadruplex configuration and prevents telomere lengthening by telomerase or by ALT [94]. This accelerates telomere shortening and subsequent cell death [95]. In addition to telomere locking property, G-quadruplex agents also interfere with telomere structure to induce telomere uncapping [96,97,98]. Several G-quadruplex stabilizers, including TMPyP4 [99,100,101], RHPS4 [102,103], BRACO-19 [104,105,106] and telomestatin [107,108,109,110,111], have been tested and induced *in vitro* cell growth arrest, increased apoptosis, *in vivo* tumor xenograft shrinkage. These are still in early development and have not entered clinical trials. Another G-quadruplex stabilizer, Quarfloxin/CX-3543, has entered phase I and II trials but is thought to induce apoptosis through inhibition of ribosomal RNA (rRNA) biogenesis [112].

T-oligo: T-oligo is an 11-mer oligonucleotide that is homologous to the 3’-telomeric overhang. Introduction of T-oligo into cancer cells mimics the presence of uncapped telomeres and induces a DNA damage response (DDR), apoptosis, and autophagy [113,114,115,116,117,118]. A study performed in melanoma cells showed that T-oligo increased p53 activity, resulting in cellular differentiation and apoptosis. In addition, the data also demonstrated that tankyrase-1, a positive telomere-lengthening regulator, is required for T-oligo mediated damage responses [118]. The mechanism of action of T-oligo targeting telomere is still under investigation, and has not yet advanced into clinical trial.

Tankyrase inhibitors: Tankyrase belongs to the poly (ADP-ribose) polymerase (PARP) protein superfamily that is involved in various cellular processes, including telomere length regulation [119]. Telomere lengthening requires dissociation of TRF1 from the telomere in order that telomerase gain access to the telomere [120]. Tankyrase facilitates TRF1 dissociation from the telomere via poly(ADP-ribosyl)ation of TRF1, followed by ubiquitin-mediated degradation of TRF1 [121,122]. Therefore, tankyrase inhibition reduces dissociation of TRF1 from the telomere and prevents binding of telomerase to telomere, thus inhibiting telomere lengthening. Tankyrase inhibition exerts additional non-telomeric effects by disrupting β-catenin-dependent transcriptional activity [123]. To date, tankyrase inhibition has been shown to enhance telomere shortening [124], and promote cell death [125]. Several tankyrase inhibitors (e.g., IWR1, IWR2, JW55, flavone, and XAV939) [126] are being tested but have not as yet entered clinical trials.

### 5.2. Direct Telomerase Inhibition

Perhaps the most intuitive telomerase-based therapeutic strategy involves direct inhibition of telomerase enzymatic activity. In 2003, the first-in-class modified oligo-nucleotide, GRN163L (Imetelstat), was developed by Geron, Inc., as a novel anticancer agent [127]. Imetelstat is a 13-mer (5’-TAGGGTTAGACAA-3’) that is complementary to nine nucleotides in the template region and four nucleotides 5’ of the template region of TERC, a targeting strategy that maximizes inhibition [128]. Modification of N3’→P5’ thio-phosphoramidate (NPS) conferred more stabilized binding of Imetelstat to its target and increased cellular and tissue penetration [127]. By directly binding the TERC template, Imetelstat disrupts telomerase ribonucleoprotein (TERT + TERC) assembly and enzymatic activity at telomeres. *In vitro*, this results in telomere shortening and eventual DNA damage and cell death. These early results in bladder, brain, breast, liver, lung, and prostate cancer prompted multiple clinical trials (Table 1). However, in trials to treat patients with non-small-cell lung cancer (NSCLC) and breast cancers, imetelstat did not meet its efficacy endpoints [128]. The main toxicities were grade 3/4 neutropenia and thrombocytopenia [129]. In subsequent trials, imetelstat was tested in patients with essential thrombocythemia (ET) and myelofibrosis (MF). In a phase 2 clinical study of imetelstat in patients with ET, 18 patients in two sequential cohorts received imetelstat, and partial inhibition of telomerase activity was confirmed in a subset of 6 patients. All patients showed hematologic responses, with 89% achieving complete hematologic response. In analyses of the three main ET biomarkers, janus kinase 2 (JAK2), calreticulin (CALR) and human c-mpl gene (MPL), rapid molecular responses were observed in seven of eight patients with JAK2 V617F mutation, and CALR and MPL mutant allele burdens also decreased by 15% to 66% [130]. In another phase 2 study of imetelstat in 33 patients with MF, seven patients (21%) had complete or partial remission, a response rate previously only achievable with allogeneic stem cell transplantation. All four patients with complete response had reversal of bone marrow fibrosis, and three of four had a molecular response [131]. Interestingly, neither of these phase II studies found a correlation between clinical responses and baseline telomere length, or between response and changes in telomere length after treatment, and could not explain why some patients, but not others, showed clinical responses. At this time, the mechanism of action of imetelstat in responders is not clearly understood and may in fact be related to the phosphorothioate modification on imetelstat, which causes binding to toll like receptor 9 (TLR9), which in turn induces a myelosuppressive effect [128].

### 5.3. Telomerase Interference

In early studies aimed at elucidating the fundamental function of telomerase RNA in *Tetrahymena*, engineered mutations in the TERC template region resulted in complementary mutations in newly-synthesized telomeres, thus demonstrating the reverse transcriptase function of telomerase [124]. Incidentally, these telomeric aberrations induced phenotypic changes (e.g., extremely large, irregular cell and nuclear shapes) and senescence, ostensibly because telomere-binding proteins could no longer effectively bind the altered telomeric sequences [153]. When extended to mammalian systems, similarly altered TERC templates introduce by lentiviral infection into cancer cells induced foci of DNA damage at telomeres and characteristic “anaphase bridges” caused by telomeric fusions, and ultimately led to cancer cell apoptosis and decreased proliferation *in vitro* and in xenograft models [154,155,156,157,158]. These effects of mutant TERC were augmented by concurrent depletion of wild-type TERC [154,158]. Although this strategy works rapidly to kill multiple cancer types, effective delivery and expression of altered TERC (451 NT) in cancer cells constitutes a challenge for this therapeutic approach.

### 5.4. TERT or TERC Promoter Driven Therapy

Increased TERT promoter activity and TERT expression is a hallmark of most cancer types. Recently a highly recurrent TERT promoter mutation (C250T) was described that creates a unique site for the binding of protein complexes containing E-twenty-six (ETS) and p52 subunit of NF-κB for enhanced TERT expression, increased telomerase activity and subsequent tumorigenesis [159]. Correction of this mutation and reduction of TERT expression may ultimately be achievable using recently developed gene editing techniques. Other therapeutic strategies have sought to exploit the active promoter-driven expression of TERT or TERC in cancer to express cytotoxic agents in target cells.

Oncolytic virus: To explore a telomerase-specific oncolytic therapy, a type 5 adenovirus was constructed by inserting adenovirus E1A and E1B genes under the control of the human TERT promoter. Upon infection, E1A and E1B are expressed and induce viral replication and cellular lysis in TERT promoter active tumor cells, while sparing TERT promoter inactive benign cells [160]. *In vitro*, the oncolytic virus OBP-301 (telomelysin) has been shown to selectively lyse lung cancer cells [161], kill CD133+ human gastric cancer stem-like cells [162], and inhibit lung tumor xenografts treated with direct intratumoral injection [163]. In phase I testing, OBP-301 was well tolerated at three different dose levels with no serious adverse events [136]. Viral DNA was detected in plasma in 13 of 16 patients, and one patient experienced a partial response at the injected malignant lesion [136]. Currently, OBP-301 is in a phase1/2 study in patients with hepatocellular carcinoma (Table 1).

Suicide gene therapy: This strategy exploits the highly active TERT or TERC promoter in tumor cells to express cytotoxic products. An adenoviral system was constructed by engineering the bacterial nitroreductase (NTR) under the control of either the TERT or TERC promoter [164]. NTR produced by TERT/TERC active cancer cells then converts the pro-drug CB1954 into active cytotoxic 2- and 4-hydroxylamino derivatives that form DNA crosslinks via an N-acetoxy intermediate. This approach induced cell death in various cancer cell lines and significant tumor reduction in xenograft models [165]. In a similar approach, carboxypeptidase G2 (CPG2) was inserted into an adenovirus expressing under the control of the human TERT promoter. Upon delivery, tumor specific expression of CPG2 converts a prodrug ZD2767P into a DNA-damaging alkylating drug ZD2767 [166]. The oncolytic effect of adenovirus with an empty construct did not cause apoptosis, while application of adenovirus with TERT-CPG2 cassette resulted in cancer cell death and tumor shrinkage in several carcinoma cell lines and in a SW620 xenograft model [166]. In a third study, a lentiviral system was also used to deliver a TERT promoter driven cytosine deaminase (CD) gene, whose production converts prodrug 5-flucytosine (5-FC) into 5-fluorouracil (5-FU), which is then converted to either 5-fluorodeoxyuridylic acid monophosphate to interfere with DNA synthesis, or 5-fluorouridine triphosphate to disrupt protein synthesis [167]. In combination with 5-FC, significant cell kill was observed in various cancer cell lines infected with lentivirus overexpressing the CD gene, and tumor growth in nude mice was drastically inhibited by intra-tumor injection of lentiviruses [167]. None of such suicide gene therapies have moved into clinical trial yet.

### 5.5. Telomerase Immunotherapy

Telomerase immunotherapy aims to exploit the relatively high expression of telomerase in cancer cells as a tumor neo-antigen to direct immune mediated cell kill. In benign cells, the protein component of telomerase, TERT, predominantly localizes to the nucleus and is not present on cell membrane. In cancer cells, TERT-derived peptides are processed and presented on tumor cells’ surface in the context of major histocompatibility complex (MHC) class I molecules. Even in telomerase positive cancer cells, there are only several hundred TERT proteins in a cell [168], a challenge for immunotherapy. Nevertheless, efforts have been made to demonstrate that TERT is a tumor-associated antigen (TAA) capable of triggering antitumor CD8+ cytotoxic T lymphocyte (CTL) response in multiple tumor types [169]. Telomerase based immunotherapy can be divided into two approaches: (1) direct immune activation *in vivo* or (2) *ex vivo* activation and expansion of immune cells.

Direct activation using TERT derived peptide-GV1001: GV1001 consists of 16 amino acids (p611-EARPALLTSRLRFIPK-p626) and is recognized by both MHC class I and class II molecules, thus offering the advantage of eliciting both CD8+ and CD4+ responses [138,170]. Several clinical trials have been conducted in non-small cell lung cancer (NSCLC), pancreatic cancer, hepatocellular carcinoma, and malignant melanoma (Table 1). No serious adverse events were observed in patients treated with GV1001, with mostly grade 1 or 2 injection site reaction [137]. In one phase I/II NSCLC study, GV1001-specific immune responses were observed in 13 out of 24 patients, four long-time survivors had durable GV1001-specific T-cell memory responses, and two were free of disease after 108 and 93 months, respectively [171]. However, a phase III trial combining GV1001 with gemcitabine and capecitabine in locally advanced or metastatic pancreatic cancer did not improve overall survival [141]. Another phase III study of GV1001 in combination with chemoradiotherapy in inoperable stage III NSCLC is ongoing.

Direct activation using TERT derived peptides-Vx-001: Vx-001 is composed of two peptides: a native cryptic peptide (TERT572) and an optimized variant (TERT572Y). The first vaccination of immunogenic TERT572Y initiates antitumor immune responses, followed by vaccination with native non-immunogenic TERT572 to select highly specific cytotoxic T lymphocytes against TERT572 peptide that is naturally presented by tumor cells [172]. Clinically, Vx-001 was shown to be safe and well tolerated with only local skin reactions [173]. Sixteen out of 21 patients were positive for TERT572-specific CD8+ cells after 2nd vaccination, and 10 out of 11 were positive after 6th vaccination. The estimated median overall survival (OS) was 30 months for patients with immunological responses *vs.* four months for those without immunological responses [174]. Currently, Vx-001 development is ongoing in a randomized phase IIb trial to evaluate efficacy and survival rate at 12 months in patients with NSCLC [142].

Direct activation using TERT derived peptides-GX-301: GX-301 is composed of four TERT-derived peptides, p540–548, p611–626, p672–686, and p766–780 shown to elicit T cell activation and cytokine release by ELISpot and FACS, and these immune responses were augmented when all four peptides were used in combination [175]. In a small phase I/II clinical trial in patients with prostate and renal cell carcinoma, intra-dermal GX-301 injection was well tolerated. Fourteen out of 14 (100%) patients tested positive for TERT-specific immunological responses, and four out of 14 patients had disease stabilization [176]. Currently, a phase II randomized clinical trial of GX301 vaccination is ongoing in men with castration-resistant metastatic prostate cancer (www.clinicaltrial.gov).

*Ex vivo* activation and expansion of immune cells using GRNVAC1: In this autologous vaccination approach, patient-derived dendritic cells are isolated and transfected *ex vivo* with mRNA encoding a chimeric protein (lysosomal targeting signal LAMP fused to TERT to enhance TERT peptide digestion and display), then administered to patients through intradermal injections [151]. This approach triggered TERT-specific CD4+ and CD8+ T cell responses, and treatment was well tolerated with only grade 1 toxicities. Immunized patients developed significantly higher frequencies of TERT specific CD4+ T cells, and 19 out of 20 patients were positive for TERT-specific CD8+ T cells [151]. A phase II study evaluating the safety, feasibility and efficacy of GRNVAC1 (now renamed AST-VAC1) was completed in acute myeloid leukemia (AML) patients in first complete remission (CR1) or in second complete remission (CR2) with intermediate or high-risk cytogenetics or in early relapse with <20% marrow blasts. Eleven of 19 patients developed TERT specific T cell responses, and 57% of patients over 60 years old were in complete remission at median follow-up of 52 months [147]. In a related project (AST-VAC2) the LAMP-TERT chimeric mRNA is transfected into human embryonic stem cells that are subsequently differentiated into dendritic cells in an effort to achieve greater scalability and reproducibility. AST-VAC2 is moving into a phase I/IIA clinical trial in patients with non-small cell lung cancer (NSCLC) in the UK.

## 6. Conclusions

The unique function of telomerase, predominantly in most cancer cells but minimally in normal somatic cells, offers unique diagnostic, prognostic, and therapeutic opportunities that have been intensely explored over the past two decades with the goal of leveraging telomerase dependence as an “Achilles heel” for tracking and killing cancer cells. Currently, multiple approaches are being pursued, each characterized by its own potential benefits and technical challenges. Several of these efforts have begun to bear fruit in recent clinical trials and are poised to contribute meaningfully to renewed “Moonshot” efforts to prevent and cure cancer. Still, more than 30 year after the discovery of telomerase, it is sobering to consider that despite all the biological insights accumulated regarding its central role in carcinogenesis, an effective telomerase-based cancer therapeutic has yet to be approved for clinical use. In some cases (e.g., imetelstat) an insufficient therapeutic index was achieved, and perhaps other inhibitors can now be identified using novel *in silico* library screens and improved knowledge of telomerase structure. In others (e.g., G-quadruplex inhibitors) the biological targets themselves have been elucidated only in recent years, and candidate therapeutics are in early development. In still other cases (e.g., promoter-driven genes and immunotherapies), the strategies were sound but had to await the emergence of better technologies. This is illustrated by the explosive preclinical and clinical progress recently made with immune checkpoint blockade, wherein therapeutic antibodies are used to block cancer cells from engaging cytotoxic T-Lymphocyte antigen 4 (CTLA-4) or programmed death 1 (PD-1) receptors on T lymphocytes. Interestingly, combination of checkpoint blockade with more traditional cancer vaccines has demonstrated enhanced antitumor activity in some studies, e.g., increased frequency of effector T cells in melanoma patients [177] or elevated effector T-cell infiltration in patients with pancreatic tumors [178]. Currently, there is no registered clinical trial studying the combination of telomerase vaccines with checkpoint blockade, and the efficacy and toxicity of such an approach remains to be studied. Even when they achieve efficacy, improved telomerase-based therapies will undoubtedly generate selective pressure favoring adaptive resistance mechanisms. For example, ALT and telomerase can coexist to maintain telomere length [179,180], and it is not unlikely that telomerase-targeting therapeutics may favor the emergence of ALT-dependent cancer cell survival and proliferation, posing new therapeutic challenges. Indeed, the discovery of telomerase has not yet delivered upon its promise of a cancer cure, and telomerase-based therapies may yet engender new resistant cancer phenotypes. However, despite these cautionary notes, it is undeniable that the study of telomerase has immeasurably enhanced our understanding of cancer biology and has opened new therapeutic avenues that may yet greatly impact cancer treatment.

## Figures and Tables

**Table 1:** Telomerase therapeutics currently in clinical development.

Agent	NCT Identifier	Trial	Status	Results
**Telomerase Inhibitor**				
Imetelstat	NCT01256762	Ph II: Breast cancer	Completed	N/A
Imetelstat ^[129]^	NCT01137968	Ph II: NSCLC	Completed	Failed to improve PFS; Grade 3/4 neutropenia and thrombocytopenia
Imetelstat	NCT00124189	Ph I: CLD	Completed	N/A
Imetelstat	NCT00510445	Ph I: Lung cancer	Completed	N/A
Imetelstat	NCT00718601	Ph I: Melanoma	Completed	N/A
Imetelstat ^[132]^	NCT00310895	Ph I: Solid tumor	Completed	Thrombocytopenia at doses >3.2 mg/kg/wk
Imetelstat	NCT01265927	Ph I: Breast neoplasms	Completed	N/A
Imetelstat ^[133]^	NCT00732056	Ph I: Breast cancer	Completed	No DLTs, Cytopenias
Imetelstat ^[130]^	NCT01243073	Ph II: ET	Completed	Rapid and durable hematologic and molecular responses
Imetelstat	NCT00594126	Ph I: Myeloma	Completed	N/A
Imetelstat	NCT01242930	Ph II: Myeloma	Completed	N/A
Imetelstat	NCT01273090	Ph I: Solid tumor or Lymphoma	Completed	N/A
Imetelstat	NCT01836549	Ph II: Brain tumor	Active, not recruiting	N/A
Imetelstat ^[131,134,135]^	NCT01731951	Ph II: Myelofibrosis	Active, not recruiting	Complete or partial remissions with various toxicity
Imetelstat	NCT02598661	Ph III: MDS	Recruiting	N/A
Imetelstat	NCT02426086	Ph II: Myelofibrosis	Recruiting	N/A
**Oncolytic Virus**				
Telomelysin/OBP-301 ^[136]^	NCT02293850	Ph I/II: HCC	Recruiting	Pain at injection site, fevers, chills; detected viral DNA in 13/16 pts
**Telomerase Vaccine**				
GV1001	NCT01223209	Ph I: Carcinoma	Recruiting	N/A
GV1001 ^[137]^	NCT00444782	Ph II: HCC	Completed	Well tolerated, mild injection site reaction; No antitumor immune response
GV1001 ^[138]^	NCT00509457	Ph I/II: NSCLC	Completed	Minor side effects, no bone marrow toxicity; Immune response in 13/24 pts
GV1001 ^[139,140]^	NCT01247623	Ph I/II: Melanoma	Completed	Well tolerated with neutropenia in 1/14 pts; Immune response in 17/21 pts; highly diverse TERT-specific T-cell response
GV1001 ^[141]^	NCT00358566	Ph III: Pancreatic cancer	Terminated	No survival benefit
GV1001	NCT00425360	Ph III: Pancreatic cancer	Completed	N/A
GV1001	NCT01342224	Ph I: Pancreatic adenocarcinoma	Active, not recruiting	N/A
GV1001	NCT01579188	Ph III: NSCLC	Not yet recruiting	N/A
VX-001 ^[142]^	NCT01935154	Ph II: NSCLC	Active, not recruiting	
GX-301	NCT02293707	Ph II: Prostate cancer	Recruiting	N/A
hTERT 540–548 peptide ^[143]^	NCT00021164	Ph II: Melanoma, Solid tumor	Completed	No immune response against hTERT + tumor
hTERT 540–548 peptide	NCT00069940	Ph I: Brain tumor, Sarcoma	Completed	N/A
hTERT 540–548 peptide ^[144]^	NCT00079157	Ph I: Breast cancer	N/A	Injection site related erythema, pain, pruritus, or induration; Prolonged survival
hTERT multi peptide ^[145]^	NCT00499577	Ph I/II: Myloma, plasma cell neoplasm	Completed	Mild to moderate chills and rigors; Antitumor immunity in 10/28 pts
hTERT multi peptide ^[146]^	NCT00573495	Ph I: Breast cancer	Completed	Well tolerated; Immune response in 80% pts
hTERT multi peptide	NCT00834665	Ph I/II: Myeloma	N/A	N/A
GRNVAC1/AST-VAC1 ^[147]^	NCT00510133	Ph II: AML	Completed	TERT specific T cell response in 11/19 pts; 57% pts in complete remission at follow-up 52 months
AST-VAC2	N/A	Ph I/II: NSCLC	N/A	N/A
DC pulsed with hTERT 540–548 peptide ^[148]^	N/A	Ph I: Breast and Prostate cancer	N/A	Well tolerated, no changes in B cells number; Immune response in 4/7 pts
DC pulsed with Telomerase peptide or tumor lysates ^[149]^	NCT00197912	Ph I/II: Melanoma	Completed	Feasible and safe: Prolonged survival
DC pulsed with Telomerase peptide or tumor lysates ^[150]^	NCT00197860	Ph I/II: RCC	Completed	Well tolerated without severe toxicities; Disease stabilized in half pts
DC pulsed with hTERT mRNA ^[151]^	NCT01153113	Ph I/II: Prostate cancer	Withdrawn	Fatigue or flu-like symptoms, erythema/induration; Immune response in 19/20 pts
TLI (hTERT DNA fragment) ^[152]^	NCT00061035	Ph I: Prostate cancer	Completed	Feasible and safe: Immune response by single-dose TLI

AML = acute myeloid leukemia; CLD = chronic lymphoproliferative disease; DC = dendritic cell; LT = dose-limiting toxicity; ET = essential thrombocythemia; HCC = hepatocellular carcinoma; MDS = myelodysplastic syndrome; N/A = not available; NCT = National Clinical Trials; NSCLC = non-small-cell lung cancer; PFS = progression-free survival; Ph = phase; pts = patients; RCC = renal cell carcinoma; TLI = transgenic lymphocyte immunization.
